# Zoonotic Virus Seroprevalence among Bank Voles, Poland, 2002–2010

**DOI:** 10.3201/eid2508.190217

**Published:** 2019-08

**Authors:** Maciej Grzybek, Tarja Sironen, Sanna Mäki, Katarzyna Tołkacz, Mohammed Alsarraf, Aneta Strachecka, Jerzy Paleolog, Beata Biernat, Kaludiusz Szczepaniak, Jolanta Behnke-Borowczyk, Antti Vaheri, Heikki Henttonen, Jerzy M. Behnke, Anna Bajer

**Affiliations:** Medical University of Gdansk, Gdansk, Poland (M. Grzybek, B. Biernat);; University of Helsinki, Helsinki, Finland (T. Sironen, S. Mäki, A. Vaheri);; University of Warsaw, Warsaw, Poland (K. Tolkacz, M. Alsarraf, A. Bajer);; University of Life Sciences in Lublin, Lublin, Poland (A. Strachecka, J. Paleolog, K. Szczepaniak);; Poznan University of Life Sciences, Poznan, Poland (J. Behnke-Borowczyk);; Natural Resources Institute Finland, Helsinki (H. Henttonen);; University of Nottingham, Nottingham, UK (J.M. Behnke)

**Keywords:** arenavirus, hantavirus, cowpox virus, Myodes glareolus, intrinsic factors, extrinsic factors, seromonitoring, seroprevalence, long-term approach, zoonoses, bank voles, Poland, viruses

## Abstract

Bank voles in Poland are reservoirs of zoonotic viruses. To determine seroprevalence of hantavirus, arenavirus, and cowpox virus and factors affecting seroprevalence, we screened for antibodies against these viruses over 9 years. Cowpox virus was most prevalent and affected by extrinsic and intrinsic factors. Long-term and multisite surveillance is crucial.

The most prevalent rodentborne zoonotic viruses in Europe are hantaviruses, lymphocytic choriomeningitis virus (LCMV), cowpox virus (CPXV), and Puumala virus (PUUV) (*1*). In 2016, a total of 18 countries in Europe reported 2,190 cases of hantavirus disease, mainly caused by PUUV. The occurrence of rodentborne viruses in Poland is not well documented. The first outbreak of hantavirus infections among humans (9 cases) was reported in 2007. During 2012–2016, a total of 79 cases of hantavirus infections were reported in Poland, 55 of them in Podkarpackie Province in 2014 (*2*). In 2015, a case of human cowpox infection was reported in Poland (*3*).

We conducted a multisite, long-term study of hantavirus and arenavirus seroprevalence in northeastern Poland. Our objectives were to monitor seroprevalence of LCMV, CPVX, and PUUV in 3 populations of bank voles (*Myodes glareolus*) from ecologically similar but disparate sites in northeastern Poland and to analyze intrinsic (host sex, host age) and extrinsic (study year, study sites) factors that might affect seroprevalence among these rodent populations.

Study sites were located in the Mazury Lake District region in northeastern Poland (Appendix Figure 1). The sites and methods used for trapping rodents and sampling and processing trapped animals have been described (*4*). We analyzed serum samples by using an immunofluorescence assay (IFA) (Appendix Figure 2). We diluted serum samples 1:10 in phosphate-buffered saline and tested their reactivity to hantaviruses by using a PUUV IFA, to cowpox viruses by using a CPXV IFA, and to arenaviruses by using an LCMV IFA (*5*). IFAs were conducted as previously described (*6*,*7*). The statistical approach has been comprehensively documented (*4*).

We tested 652 bank voles and detected antibodies against all 3 viruses. Overall seroprevalence of combined viral infections was 25.9% (95% CI 23.0%–29.1%), but most infections were attributable to CPXV (seroprevalence 25% [95% CI 22.1%–28.2%]). Only 2 voles were LCMV seropositive (0.3% [95% CI 0.2%–0.9%]), and only 5 were PUUV seropositive (0.76% [95% CI 0.4%–1.6%]). We therefore confined further analyses to CPXV.

The effect of study year on CPXV seroprevalence (by χ2/d.f.) was highly significant (χ^2^_2_ 31.2; p<0.001); seroprevalence was 2.7 times higher among bank voles sampled in 2010 (36.1% [95% CI 31.7%–40.7%]) than in 2002 (13.1% [7.4%–21.0%]). CPXV seroprevalence also varied markedly among voles from the 3 study sites (χ^2^_2_ 46.84; p<0.001); seroprevalence was highest among voles from Urwitałt (38.4% [95% CI 33.9%–43.1%]) and lower among voles from Tałty (23.0% [19.3%–27.2%]) and Pilchy (10.3% [95% CI 5.7%–17.4%]). CPXV seroprevalence was also significantly affected by the sex of the host (χ^2^_1_ 10.1; p = 0.001) and was 1.5 times higher for male than female voles (Figure, panel A). Seroprevalence increased with host age (χ^2^_2_ 12.73; p = 0.002) and was lowest among voles from age class 1 (immature) (16.0% [95% CI 10.0%–24.1%]) and higher among those from age class 2 (mostly young adults) (27.2% [95% CI 23.2%–31.5%]) and age class 3 (breeding older adults) (30.1% [95% CI 25.9%–34.6%]).

**Figure:**
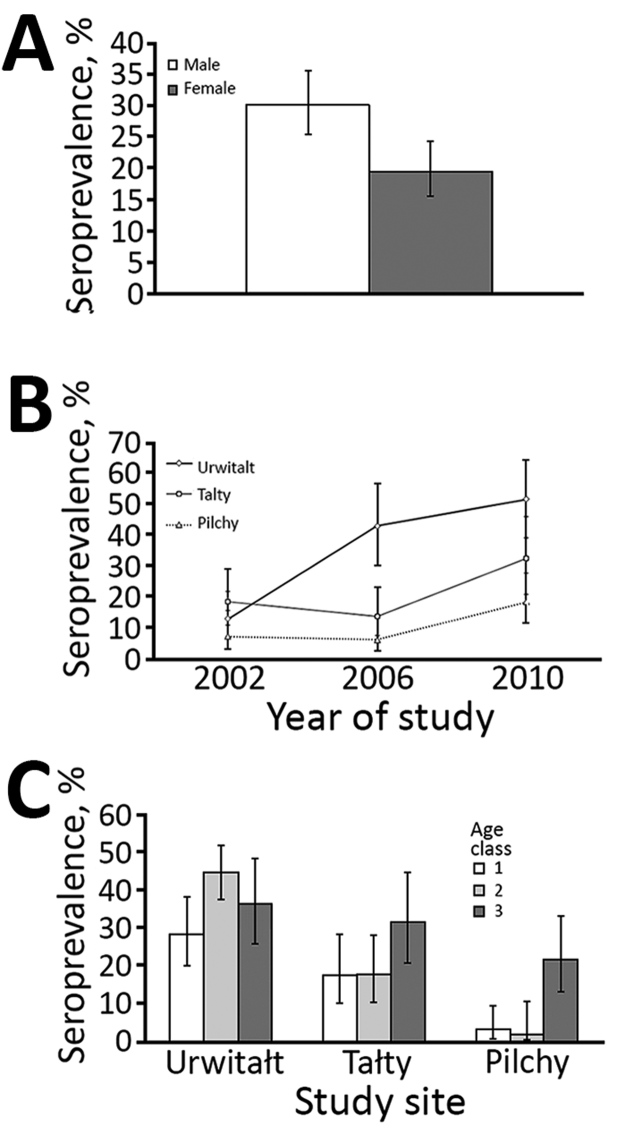
Seroprevalence of cowpoxvirus (CPXV) in bank voles in Poland, 2002–2010. A) By sex; B) by study site location and year of study; C) by study site location and vole age class (class 1—immature juvenile bank voles; class 2—mostly young adult bank voles; and class 3—breeding older animals). Error bars indicate 95% CI.

The differences in seroprevalence between sites were also confounded by interaction with study year (year × site × presence/absence of antibodies against CPXV; χ^2^_4_ 12.76; p = 0.012). Seroprevalence increased significantly at all 3 study sites from 2006 to 2010 and was highest in Urwitałt (0.83-fold). The largest seroprevalence increases from 2006 to 2010 were in Tałty (2.35-fold) and Pilchy (2.9-fold) (Figure, panel B).

The pattern of age-related changes in seroprevalence also differed between study sites (site × age × presence/absence of antibodies against CPXV; χ^2^_4_ = 17.45; p = 0.002) (Figure, panel C). In Urwitałt, the overall seroprevalence was highest among voles in age class 2 (44.5% [95% CI 37.5%–51.8%]), 1.57-fold lower among voles in age class 1, and 1.22-fold lower among voles in age class 3. In Tałty and Pilchy, seroprevalence was highest among voles from age class 3. In Tałty, seroprevalence was 1.8-fold higher among voles in age class 3 compared with voles in other age classes. In Pilchy, seroprevalence among voles in age class 3 was 10.8-fold higher than among voles in age class 2.

Our data show that CPXV was the dominant viral pathogen among bank voles in Poland during the study period, although PUUV and LCMV were also found. Our finding that the highest seroprevalence was among bank voles from Urwitałt complements our previous reports on other pathogens, reflects the importance of extrinsic effects on prevalence, and establishes that the sites from which host populations are sampled is the most influential factor affecting prevalence (*4*).

Our results provide additional information about the role of bank voles in Poland as infectious virus reservoirs. Although short-term cross-sectional studies are useful as a starting point (*8*), to obtain a comprehensive ecologic picture, long-term monitoring (several years and preferably a decade or longer) and a multisite approach are crucial. Identifying rodent species that can serve as reservoirs for zoonotic disease viruses and predicting regions where new outbreaks are most likely to happen are crucial steps for preventing and minimizing the extent of zoonotic disease among humans (*9*).

**Appendix.** Study sites and test results from study of zoonotic virus seroprevalence among bank voles, Poland, 2002–2010.
