# Supplementary material for: Zoonotic Virus Seroprevalence among Bank Voles, Poland, 2002–2010
Source: Emerg Infect Dis. 2019 Aug;25(8):1607–9. doi: 10.3201/eid2508.190217 (PMC6649315; doi:10.3201/eid2508.190217)
Supplement: Supplementary file 1 — Appendix. Study sites and test results from study of zoonotic virus seroprevalence among bank voles, Poland, 2002–2010. [file 19-0217-Techapp-s1.pdf]

Article DOI: https://doi.org/10.3201/eid2508.190217

Zoonotic Virus Seroprevalence among Bank Voles, Poland, 2002–2010

Appendix

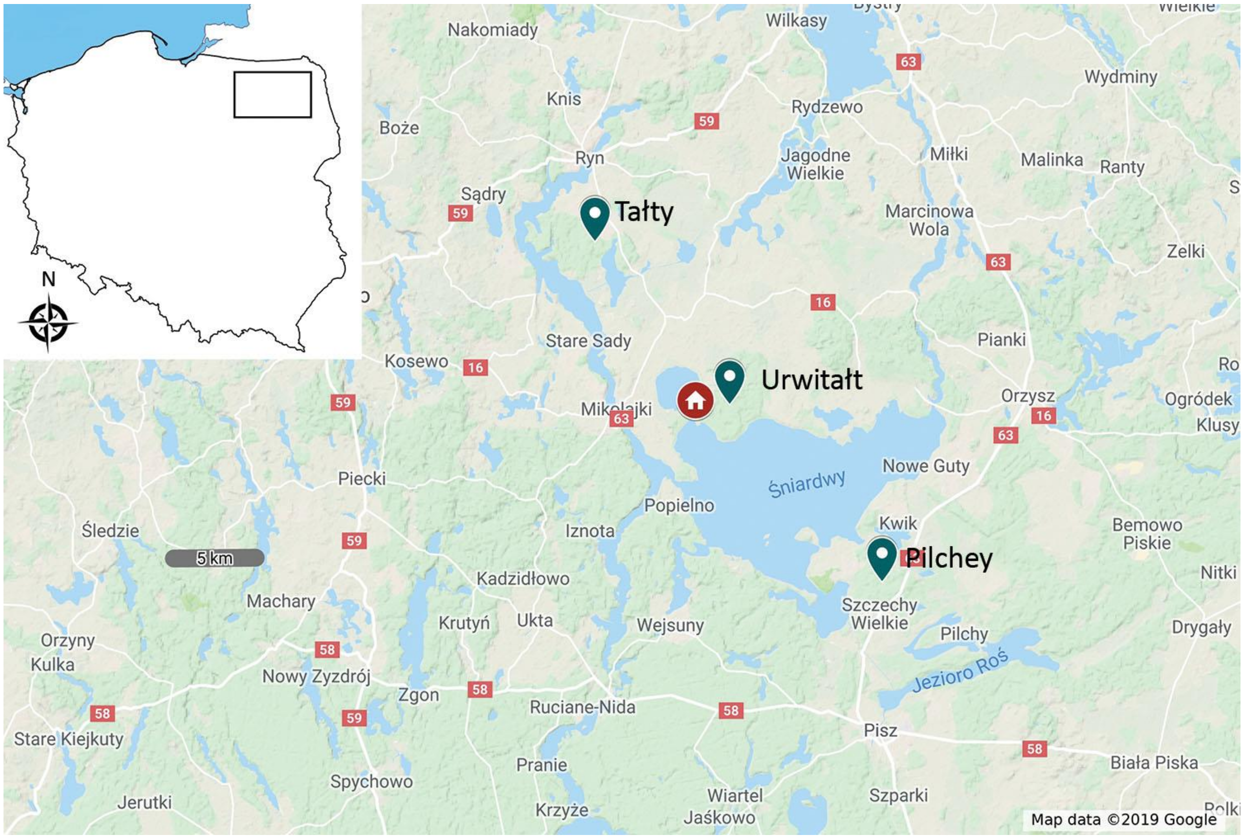

Appendix Figure 1. Locations of study sites in the Mazury Lake District in northeastern Poland. Red pointers, study sites. Blue pointer; Warsaw University Field Station. Source: Google Maps.

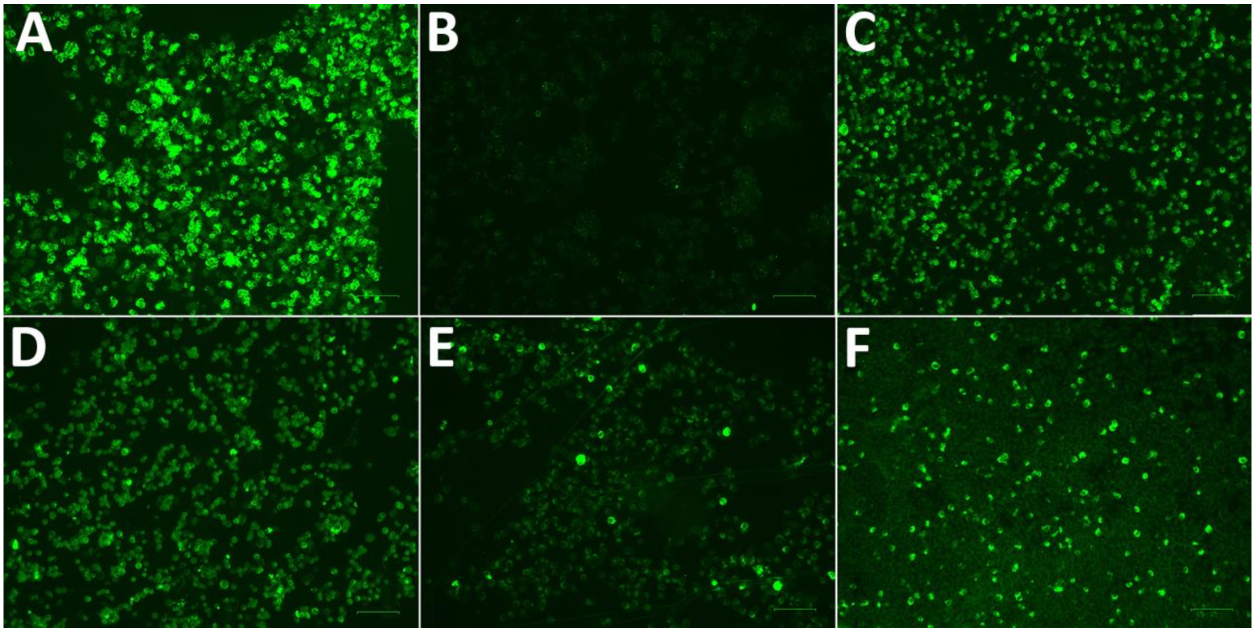

Appendix Figure 2. Immunofluorescence assay (IFA) for: PUUV (A) positive control, (B) positive sample; LCMV (C) positive control, (D) positive sample; CPXV (E) positive control, (F) positive sample. The serum samples were diluted 1:10 in PBS, and the reactivity of the samples to hantaviruses were tested with PUUV-IFA, to cowpox viruses with CPXV-IFA and arenaviruses with LCMV-IFA. Briefly, PUUV (Sotkamo strain), CPXV, and LCMV (Armstrong strain)-infected Vero E6 cells were detached with trypsin, mixed with uninfected Vero E6 cells (in a ratio of 1: 3), washed with PBS, spotted on IFA slides, air-dried, and fixed with acetone as described earlier (*1*). The slides were stored at –70°C until use. IFAs were carried out with seropositive human serum as a positive control for the PUUV- and CPXV-IFA; and LCMV mouse monoclonal antibody (Progen, Heidelberg) for the LCMV-IFA. The slides were read under a fluorescence microscope, and pictures were taken with a ZOE fluorescent cell imager (BioRad).

Reference

1. Hedman K, Vaheri A, Brummer-Korvenkontio M. Rapid diagnosis of hantavirus disease with an IgG-avidity assay. Lancet. 1991;338:1353–6.
